# Antimicrobial Susceptibility of *Flavobacterium psychrophilum* from Chilean Salmon Farms and Their Epidemiological Cut-Off Values Using Agar Dilution and Disk Diffusion Methods

**DOI:** 10.3389/fmicb.2016.01880

**Published:** 2016-11-25

**Authors:** Claudio D. Miranda, Peter Smith, Rodrigo Rojas, Sergio Contreras-Lynch, J. M. Alonso Vega

**Affiliations:** ^1^Laboratorio de Patobiología Acuática, Departamento de Acuicultura, Universidad Católica del NorteCoquimbo, Chile; ^2^Centro AquaPacíficoCoquimbo, Chile; ^3^Department of Microbiology, School of Natural Sciences, National University of IrelandGalway, Ireland; ^4^Departamento de Salud Hidrobiológica, Instituto de Fomento PesqueroPuerto Montt, Chile; ^5^Programa Consorciado Doctorado en Acuicultura, Universidad Católica del NorteCoquimbo, Chile

**Keywords:** *Flavobacterium psychrophilum*, epidemiological cut-off value, MIC, fish pathogen, antimicrobial susceptibility, Chile

## Abstract

*Flavobacterium psychrophilum* is the most important bacterial pathogen for freshwater farmed salmonids in Chile. The aims of this study were to determine the susceptibility to antimicrobials used in fish farming of Chilean isolates and to calculate their epidemiological cut-off (CO_WT_) values. A number of 125 Chilean isolates of *F. psychrophilum* were isolated from reared salmonids presenting clinical symptoms indicative of flavobacteriosis and their identities were confirmed by 16S rRNA polymerase chain reaction. Susceptibility to antibacterials was tested on diluted Mueller-Hinton by using an agar dilution MIC method and a disk diffusion method. The CO_WT_ values calculated by Normalized Resistance Interpretation (NRI) analysis allow isolates to be categorized either as wild-type fully susceptible (WT) or as manifesting reduced susceptibility (NWT). When MIC data was used, NRI analysis calculated a CO_WT_ of ≤0.125, ≤2, and ≤0.5 μg mL^-1^ for amoxicillin, florfenicol, and oxytetracycline, respectively. For the quinolones, the CO_WT_ were ≤1, ≤0.5, and ≤0.125 μg mL^-1^ for oxolinic acid, flumequine, and enrofloxacin, respectively. The disk diffusion data sets obtained in this work were extremely diverse and were spread over a wide range. For the quinolones there was a close agreement between the frequencies of NWT isolates calculated using MIC and disk data. For oxolinic acid, flumequine, and enrofloxacin the frequencies were 45, 39, and 38% using MIC data, and 42, 41, and 44%, when disk data were used. There was less agreement with the other antimicrobials, because NWT frequencies obtained using MIC and disk data, respectively, were 24 and 10% for amoxicillin, 8 and 2% for florfenicol, and 70 and 64% for oxytetracycline. Considering that the MIC data was more precise than the disk diffusion data, MIC determination would be the preferred method for susceptibility testing for this species and the NWT frequencies derived from the MIC data sets should be considered as the more authoritative. Despite the high frequency of isolates showing full susceptibility to florfenicol, the significant frequencies of isolates exhibiting reduced susceptibility to oxytetracycline and quinolones may result in treatment failures when these agents are used.

## Introduction

*Flavobacterium psychrophilum*, the causative agent of bacterial cold-water disease (CWD) and rainbow trout fry syndrome (RTFS), is a gram-negative bacterium that produces an acute septicaemic infection in salmonids (Barnes and Brown, 2011) causing significant losses to trout and salmon farming worldwide (Nematollahi et al., 2003).

The lack of an effective commercial vaccine results in antibiotic treatment being currently the treatment of choice for controlling losses resulting from *F. psychrophilum* infections (Barnes and Brown, 2011). According to these authors amoxicillin, oxytetracycline, florfenicol, oxolinic acid and various sulphonamides or potentiated sulphonamides all have been reported as being used for this purpose. Data on the current susceptibility to these agents is therefore of critical importance for the adoption of appropriate therapeutic strategies.

Many studies, from many countries, have reported on the antibiotic susceptibility of *F. psychrophilum.* However, because of the difference in methods and test protocols used in these studies it has proved difficult to combine or compare the data they generated. Some studies have employed disk diffusion (Dalsgaard and Madsen, 2000; Kum et al., 2008; Durmaz et al., 2012; Henríquez-Núñez et al., 2012) whilst others determined MIC values. The MIC studies have variously employed agar dilution (Hawke and Thune, 1992; Bruun et al., 2000; Schmidt et al., 2000; Michel et al., 2003; Izumi and Aranishi, 2004; Kum et al., 2008), broth macro-dilution (Del Cerro et al., 2010), or broth micro-dilution (Rangdale et al., 1997; Hesami et al., 2010; Henríquez-Núñez et al., 2012: Smith et al., 2016). Although most studies used diluted Mueller-Hinton (MH) media there has been variation in the extent of the dilution and the use of additives. There has also been a wide variation, from 5 (Durmaz et al., 2012) to 387 (Bruun et al., 2000) in the number of isolates included in these studies. Possibly of more significance, for the purpose of facilitating comparisons, has been the variation in the criteria, or in some studies the lack of them, used interpret the meaning of the data obtained in the various studies. The World Animal Health Organisation^1^ has recommended that epidemiological cut-off values calculated by standardized and statistically based analytical methods should be used as interpretive criteria. These species-specific cut-off values allow the categorisation of isolates either as wild-type (WT) fully susceptible members of their species or as manifesting reduced susceptibility (NWT; Silley, 2012). Only two studies (Henríquez-Núñez et al., 2012; Smith et al., 2016) have, so far, taken this approach and both used normalized resistance interpretation (NRI) method developed by Kronvall (2003, 2010).

In Chile *F. psychrophilum* is currently considered the most important bacterial pathogen in Chilean freshwater salmonid farms (Avendaño-Herrera et al., 2014), affecting rainbow trout (*Oncorhynchus mykiss*), Atlantic salmon (*Salmo salar*) and coho salmon (*O. kisutch*). In this country, control of this pathogen relies on antibiotic therapy mainly based on florfenicol and oxytetracycline usage. The failure to detect the occurrence of isolates with reduced susceptibility may result in treatment failures. Thus, acquisition of knowledge as to the antimicrobial susceptibility patterns in Chilean isolates of this highly prevalent pathogenic species is an urgent need if more rational, prudent and effective therapies are to be developed.

Currently, only two studies describing the antimicrobial susceptibility of *F. psychrophilum* isolated from Chilean farms are available, but in both cases only a low number of isolates were studied. Valdebenito and Avendaño-Herrera (2009) studied the susceptibility to antibacterials of 20 Chilean *F. psychrophilum* isolates, using an agar disk diffusion test on diluted Mueller-Hinton agar supplemented with 5% fetal calf serum. They detected a high susceptibility to amoxicillin and florfenicol, and resistance to sulfamethoxazole-trimethoprim. In a more recent study, Henríquez-Núñez et al. (2012) studied the susceptibility to florfenicol, oxytetracycline, and oxolinic acid of 40 *F. psychrophilum* isolates recovered from Chilean salmonid farms. Their MIC data indicated a high incidence of resistance to these antibacterials. However, they reported no correlations between MIC values and inhibition zones for the antimicrobials florfenicol and oxytetracycline and their results from disk diffusion assays for oxolinic acid were inconclusive.

It must be emphasized the urgent need for Chilean salmonid farms of a standardized antimicrobial susceptibility protocols and precise interpretative standards for *F. psychrophilum*, based on cut-off values obtained by using standardized methodologies to detect isolates exhibiting reduced susceptibility to antimicrobials in order to prevent therapy failures in the future.

The main goal of this study was to gain information on the susceptibility to the main groups of antimicrobials used in fish farming of *F. psychrophilum* isolates recovered from Chilean farmed salmonids presenting clinical signs of flavobacteriosis. The susceptibilities of 125 isolates were measured using both agar dilution MIC and disk diffusion protocols. NRI analysis was used to set epidemiological cut-off values for the data obtained and to evaluate the precision of the data obtained by the two protocols.

## Materials and Methods

### Bacterial Isolates

A total of 125 isolates of *F. psychrophilum* recovered from external lesions (ulcers, gill, or fin lesions) or internal organs (kidney, spleen, or pericardial cavity) exhibited by rainbow trout *O. mykiss* (96 isolates), Atlantic salmon *S. salar* (27 isolates), or coho salmon *O. kisutch* (2 isolates) positively diagnosed with flavobacteriosis sampled from various freshwater Chilean farms located in the South of Chile were studied. Isolates were isolated in the fish pathological diagnostic laboratory ADL Diagnostics, and sent to the Aquatic Pathobiology Lab of the Universidad Católica del Norte, where purified isolates were stored at -85°C in CRYOBANK^TM^ vials (Mast Diagnostica, Reinfeld, Germany) until use. The quality control strains *Aeromonas salmonicida* subsp. *salmonicida* ATCC 33658 and *Escherichia coli* ATCC 25922 were used in the antimicrobial susceptibility assays. Additionally, reference strain *F. psychrophilum* ATCC 49418 was included in all phenotypic, genotypic and antimicrobial susceptibility analyses for comparative purposes. *F. psychrophilum* ATCC 49418 was isolated in 1989 and was, therefore, presumed not to have been exposed to the same levels and range of antibacterial compounds as the Chilean isolates studied here. All isolates were recovered and cultured prior to being analyzed using the FLPA medium (Cepeda et al., 2004).

### Antimicrobial Agents

The antimicrobial agents used to perform the susceptibility assays of *F. psychrophilum* isolates were those currently licensed for treatment of Gram-negative bacterial pathogens in the Chilean salmonid farm industry. In addition, due to its worldwide use in fish aquaculture, enrofloxacin was included in the study. The antimicrobials used were amoxicillin, florfenicol, oxytetracycline, oxolinic acid, flumequine and enrofloxacin, and were commercially obtained from Sigma (Sigma, Poole, UK). Antimicrobial solutions were prepared according to CLSI guidelines (CLSI, 2006) and solvents used were deionized distilled water for oxytetracycline-HCl, 95% ethanol for amoxicillin and florfenicol and 0.1 M NaOH for oxolinic acid, flumequine, and enrofloxacin. All solutions were filter-sterilized (0.22 μm) and used immediately.

### Bacterial Identification

Isolates were identified as *F. psychrophilum* on the basis of their cultural characteristics (aerobic, glucose fermenter, oxidase producer Gram negative bacilli producing yellow-pigmented colonies and exhibiting gliding motility), and these tests were performed as described by Lorenzen et al. (1997). The identities of *F. psychrophilum* isolates were confirmed by polymerase chain reaction (PCR) using the 16S r RNA-specific primers for *F*. *psychrophilum* PSY-1 (5′-CGATCCTACTTGCGTAG-3′) and PSY-2 (5′-GTTGGCATCAACACACT-3′) as previously described (Toyama et al., 1994). The 16S rRNA gene amplicons were sequenced in Macrogen USA (Rockville, MD, USA) and nucleotide sequence alignment and comparisons were carried out by using BLAST (Basic Local Alignment Search Tool) on the NCBI (National Center for Biotechnology Information), Ribosomal Database Project (RDP^2^; Maidak et al., 1999).

### Disk Diffusion Assays

Isolates were tested for their susceptibility to the antimicrobials by an agar disk diffusion method as was essentially described in the Clinical and Laboratory Standards Institute (CLSI) guideline M42-A (CLSI, 2006), using 1:5 Diluted Cation-Adjusted Mueller-Hinton II Agar (DCAMHA, BBL-Becton Dickinson). The antibacterial susceptibility patterns of *F. psychrophilum* isolates were performed using disks containing the antibacterial agents: amoxicillin (AML, 25 μg), florfenicol (FFC, 30 μg), oxytetracycline (OT, 30 μg), oxolinic acid (OA, 2 μg), flumequine (UB, 30 μg), and enrofloxacin (ENR, 5 μg). All disks were obtained from Oxoid Ltd (Basingstoke, Hampshire, England). Bacterial isolates were suspended in sterile 0.85% saline to a turbidity to match a McFarland No. 2 standard (bioMerieux S.A.), diluted 1:20, and inoculated on the used media. Plates were incubated for 72–96 h (growth dependent) at 18°C. The control strains were incubated at 18°C for 48 h (*A. salmonicida* ATCC 33658 and *E. coli* ATCC 25922) and 72 h (*F. psychrophilum* ATCC 49418).

### Minimum Inhibitory Concentrations (MIC) Assays

The MICs of six antimicrobial agents against the *F. psychrophilum* isolates were assessed using an agar plate dilution method. A serial twofold dilution pattern of each antimicrobial agent was added into 1:5 diluted Cation Adjusted Mueller-Hinton II Agar (DCAMHA, BBL-Becton Dickinson) as to obtain final concentrations of 0.016–1,024 μg mL^-1^, of the antimicrobials amoxicillin, florfenicol, oxytetracycline, oxolinic acid, flumequine, and enrofloxacin. Bacterial suspensions were prepared in sterile 0.85% saline and triplicate plates were inoculated using a Steers replicator apparatus (Steers et al., 1959), delivering ∼10^4^ colony-forming units per spot, and incubated at 18°C for 72–96 h. The first and the last agar plates did not contain any antibiotics in order to detect possible contamination of the isolates or antibiotic carryover. MIC was defined as the lowest concentration of an agent producing absence of growth in at least two of the three plates after 72 h. Reference strains *E. coli* ATCC 25922 and *A. salmonicida* ATCC 33658 and F. *psychrophilum* ATCC 49418 were included in each plate to be used as quality control organisms for verification of MIC ranges on Diluted Mueller-Hinton agar plates.

### Statistical Analysis

Epidemiological cut-off values were calculated for the MIC and disk zone data sets by application of NRI analysis (Kronvall, 2003, 2010). The NRI method was used with permission from the patent holder, Bioscand AB, TÄBY, Sweden (European Patent No. 1383913, US Patent No. 7,465,559). A fully automatic Excel spreadsheet for performing NRI analyses of MIC data was accessed on-line^3^. The NRI analysis of disk diffusion zone sizes was performed using a modified version of the standard method of Kronvall and Smith (in press) ^3^ available at. In the standard method the first step in the analysis of zone data is the smoothing of the observational data by calculating the 4-point rolling averages. The extreme diversity of the zone sizes obtained in this work required the adoption of a modification of this step. To facilitate NRI analysis of these diverse data the adoption of an 8-point rolling mean was found necessary.

### Terminology

The acronyms ECV and ECOFF have been used by CLSI and EUCAST, respectively, for epidemiological cut-off values they have set from data generated in multiple laboratories. We follow Smith et al. (2016) in using the term CO_WT_ to indicate epidemiological cut-off values that have not been set by either of these international agencies. Silley (2012) has argued that the terms resistant and sensitive should not be used to refer to the categories identified by epidemiological cut-off values. We follow his suggestion that when isolated are categorized by epidemiological cut-off values, the term WT should be used for fully susceptible isolates and non-WT should be used to refer to isolates exhibiting reduced susceptibility.

## Results

### Quality Control

The reference strains *E. coli* ATCC 25922, *A. salmonicida* ATCC 33658, and *F. psychrophilum* ATCC 49418 were included in all susceptibility tests performed in this work. The MIC values obtained for these reference strains all lay within the acceptable ranges published by CLSI (2014b). The value of these observations as quality control measures must not, however, be exaggerated. These acceptable ranges were developed and validated for data produced by the CLSI broth micro-dilution protocol for testing *F. psychrophilum* (CLSI, 2014a). CLSI (2014b) specifically states that these acceptable ranges cannot be automatically assumed to be valid for MIC data obtained by an agar dilution method.

Acceptable ranges for reference strains have not been set for disk zone data obtained by the protocol used in this work or by any other protocol suitable for testing *F. psychrophilum*. Although they are not strictly relevant, the reference strain data obtained in this work were compared to the acceptable ranges published in the guideline M42-A by CLSI data for tests performed on Mueller Hinton agar at 22 and 28°C (CLSI, 2006). All the reference strain zone data obtained in this work were within the all the acceptable ranges specified in M42-A.

### Minimum Inhibitory Concentrations (MIC)

The precision of MIC data sets can be assessed by calculating the standard deviations of the normalized distributions of the log_2_ transformed values obtained from putative WT isolates (Smith and Kronvall, 2015a). The mean of these standard deviations for the six data sets obtained for *F. psychrophilum* in this work was 0.77 ± 0.12. The equivalent value calculated for 38 MIC data sets obtained from single laboratories and published by EUCAST^4^ was 0.81 ± 0.20. This comparison indicates that the *F. psychrophilum* six MIC data sets obtained in this work were of adequate precision.

**FIGURE 1:**
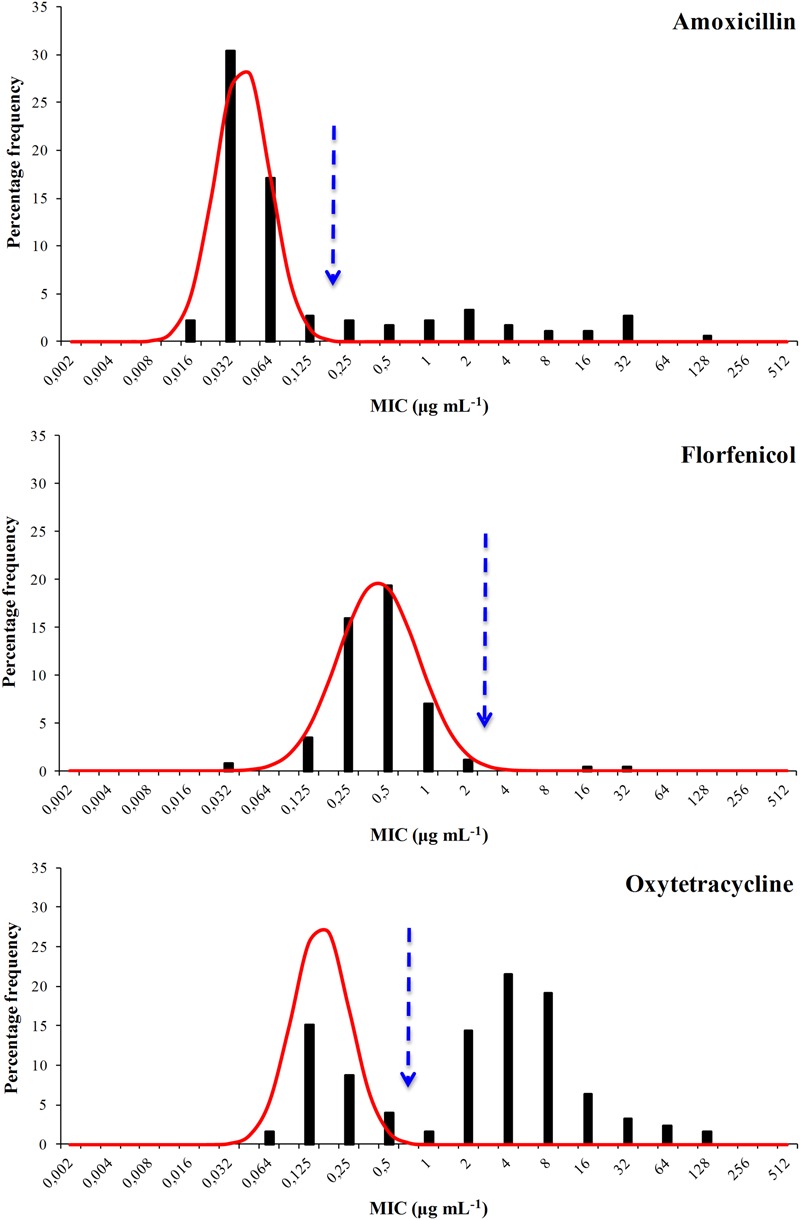
**Distribution of Minimum Inhibitory Concentrations (MIC) values of the antibacterial agents amoxicillin, florfenicol, and oxytetracycline of 125 *Flavobacterium psychrophilum* Chilean isolates.** The continuous line represents the 4-point rolling mean and the vertical dashed line the calculated epidemiological cut-off value (CO_WT_).

**FIGURE 2:**
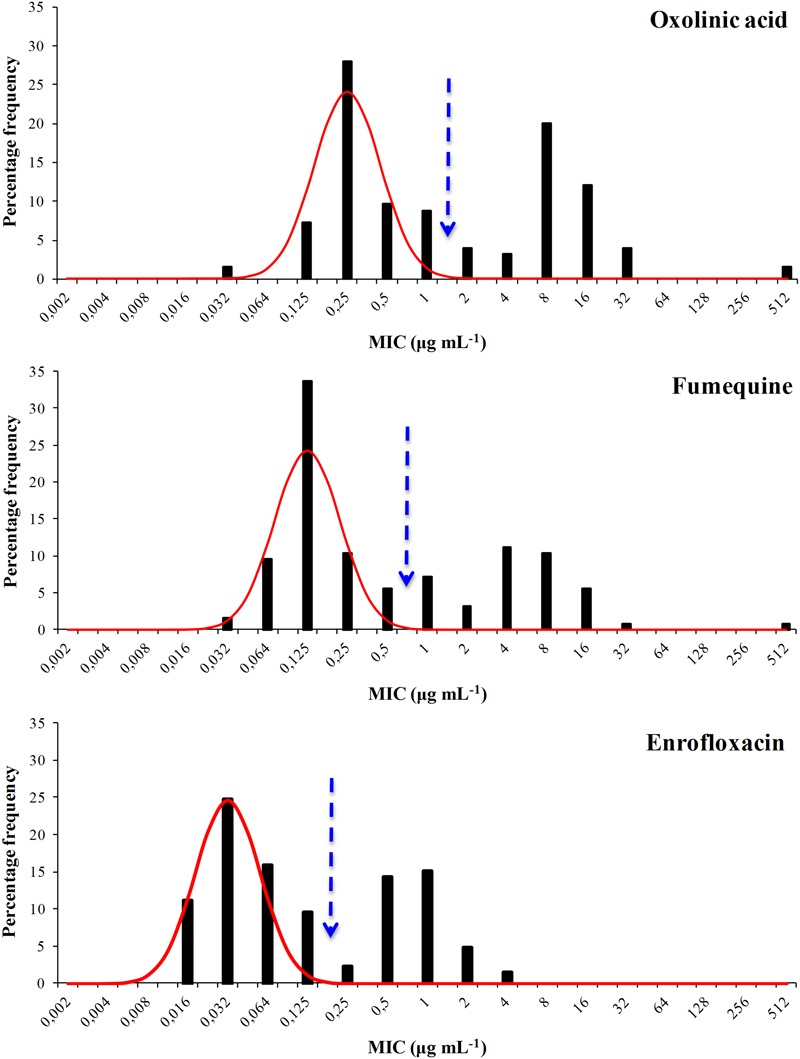
**Distribution of Minimum Inhibitory Concentrations (MIC) values of the antibacterial agents oxolinic acid, flumequine, and enrofloxacin of 125 *F. psychrophilum* Chilean isolates.** The continuous line represents the 4-point rolling mean and the vertical dashed line the calculated epidemiological cut-off value (CO_WT_).

The distribution of the MIC values for all six agents were bimodal and each showed a well defined modal group at the low MIC end (**Figures 1** and **2**) and it was assumed, for the purposes of NRI analysis, that these modal groups represented measures of fully susceptible WT members of this species. This assumption is supported by the observation that all the MIC values for the reference strain *F. psychrophilum* ATCC 49418 lay within these modal groups.

For amoxicillin NRI analysis calculated a CO_WT_ of ≤0.125 μg mL^-1^ from the MIC data. This categorized 95 isolates as WT and 30 (24%) of the isolates as manifesting reduced susceptibility. For florfenicol the CO_WT_ was ≤2 μg mL^-1^ and 123 isolates were categorized as WT. Only two isolates were categorized as manifesting reduced susceptibility. Although they had MIC values of 16 and 32 μg mL^-1^ the presence of *floR* was not detected in these isolates (Unpublished results).

For oxytetracycline the CO_WT_ was ≤0.5 μg mL^-1^. This categorized 37 isolates as WT and 88 (70%) as manifesting reduced susceptibility. For the quinolone agents the CO_WT_ were ≤1, ≤0.5, and ≤0.125 μg mL^-1^ for oxolinic acid, flumequine, and enrofloxacin, respectively. There was reasonable agreement (45% for oxolinic acid, 39% for flumequine, and 38% for enrofloxacin) between the frequencies of isolates manifesting reduced susceptibility to the three quinolones.

### Disk Diffusion Assays

**FIGURE 3:**
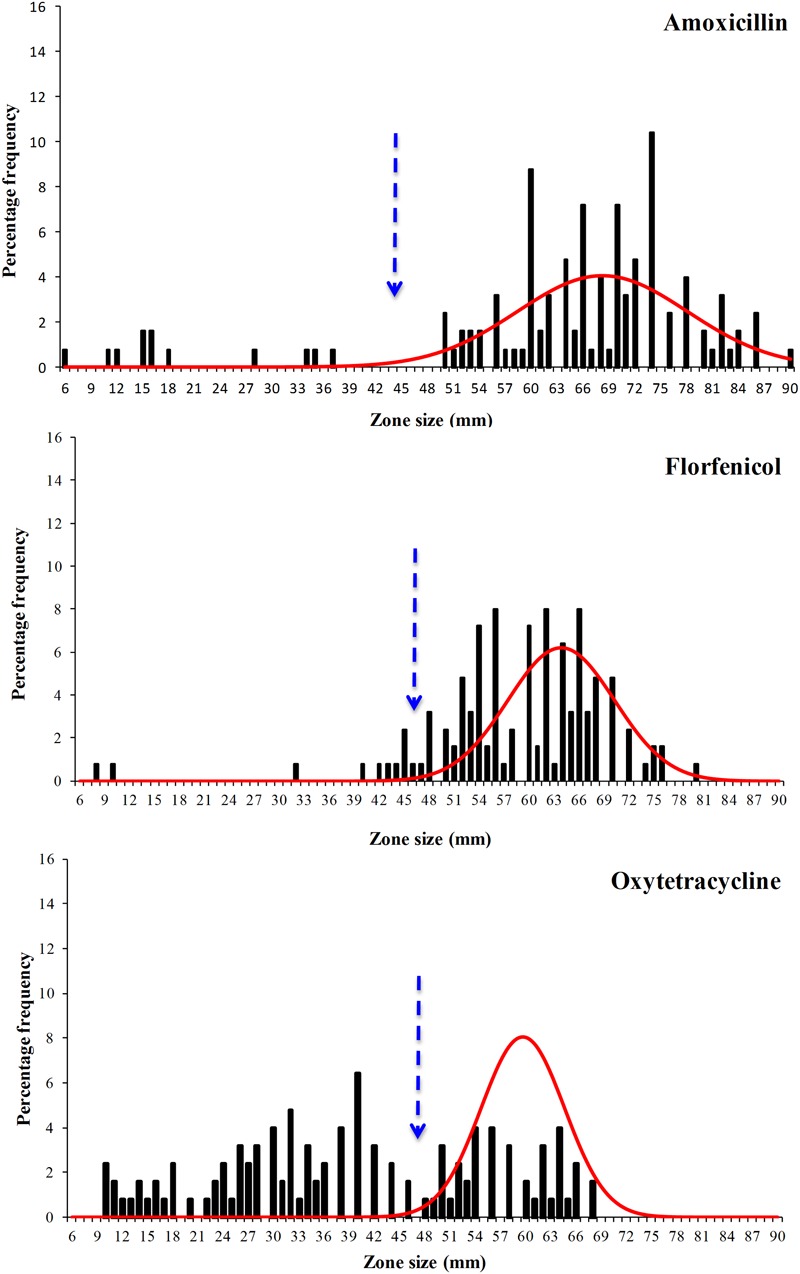
**Distribution of zones of inhibition produced by disks containing the antibacterial agents amoxicillin, florfenicol, and oxytetracycline of 125 *F. psychrophilum* Chilean isolates.** The continuous line represents the 8-point rolling mean and the vertical dashed line the calculated epidemiological cut-off value (CO_WT_).

**FIGURE 4:**
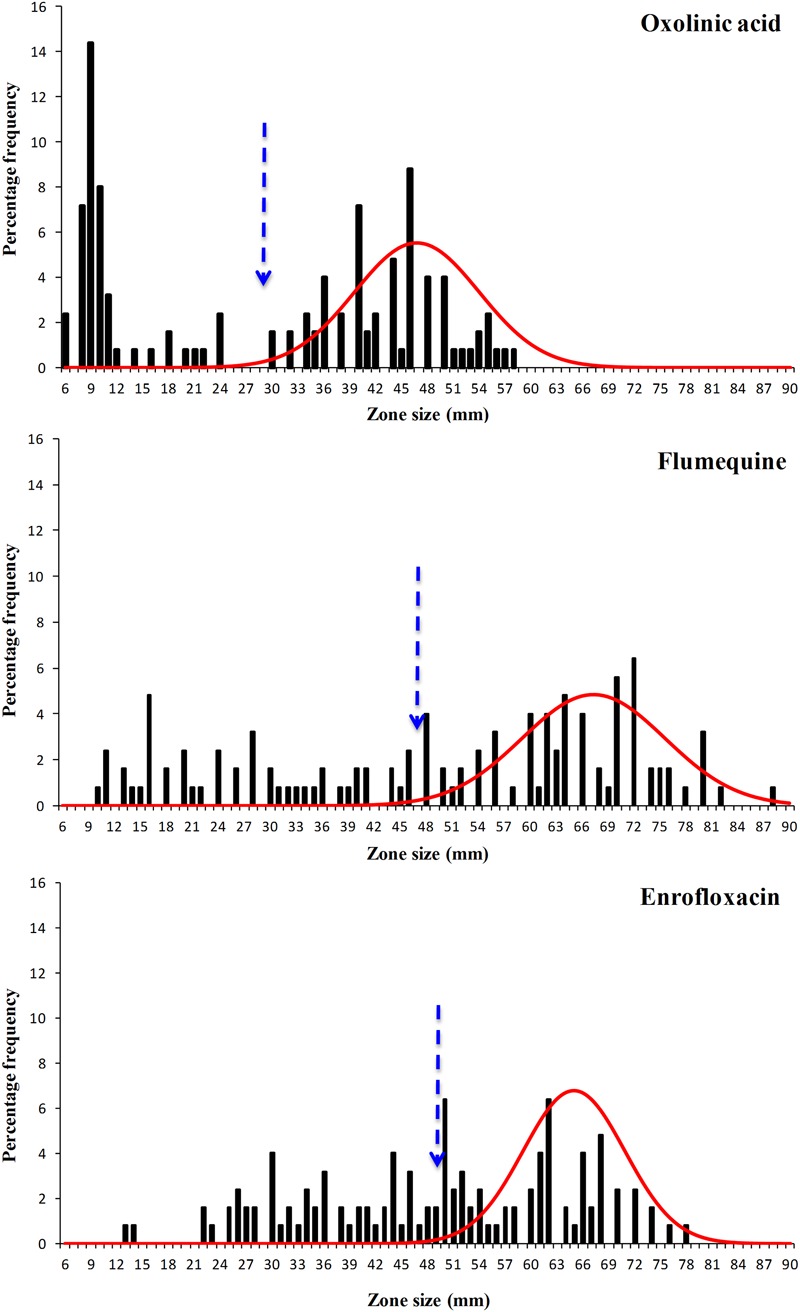
**Distribution of zones of inhibition produced by disks containing the antibacterial agents oxolinic acid, flumequine, and enrofloxacin of 125 *F. psychrophilum* Chilean isolates.** The continuous line represents the 8-point rolling mean and the vertical dashed line the calculated epidemiological cut-off value (CO_WT_).

The disk diffusion data sets obtained in this work were extremely diverse and were spread over a wide range (**Figures 3** and **4**) and visually the distributions for flumequine and enrofloxacin showed no clear high-zone modal group. Despite their diversity all six data sets showed distributions with a modal group at the high zone end and it was possible to analyse all of them using the modified version of NRI. However, the mean of the standard deviations of the normalized distributions of zone sizes obtained from the putative WT isolates generated by these NRI analyses was 7.64 mm. The comparison of this mean with the mean of 2.19 mm for 40 data sets published by EUCAST suggests that the data obtained for *F. psychrophilum* had a very low precision. Smith and Kronvall (2015a) have demonstrated that both the intra- and inter-laboratory precision that can be achieved in sets of disk diffusion zone size data decreases as the incubation temperature is reduced and the time is increased. Thus the low precision of the zone data sets for *F. psychrophilum* at 18°C for 4 days is not surprising and is probably a function of the inherent properties of disk diffusion assays. However, the low precision of the zone data sets has the automatic consequence that the accuracy of any cut-off values calculated for them is also low and that the CO_WT_ generated from them should be treated as very provisional estimates.

The provisional CO_WT_ calculated from the disk data for the six agents and the frequency of NWT phenotypes resulting from their application are shown in **Table 1**. For amoxicillin NRI analysis calculated a CO_WT_ of ≥44 mm, which categorized 113 isolates as WT and 12 (10%) of the isolates as manifesting reduced susceptibility (NWT). For florfenicol NRI analysis calculated a CO_WT_ of ≥46 mm, which categorized 8% of the isolates as NWT. For oxytetracycline NRI analysis calculated a CO_WT_ of ≥47 mm, which categorized 64% of the isolates as NWT. For the quinolones, oxolinic acid, flumequine, and enrofloxacin the CO_WT_ were calculated as ≥29, ≥47, and ≥49 mm, respectively. The frequencies of isolates categorized as NWT with respect to these three agents were very similar and ranged from 41 to 44%.

**Table 1:** Epidemiological cut-off values (CO_WT_) and the frequency of non-wild-type (NWT) phenotypes calculated by NRI analysis of MIC and disk zone data for 125 Chilean *F. psychrophilum* isolates.

Antibiotic	MIC data		Disk data	
	CO_WT_ (μg mL^-1^)	NWT (%)	CO_WT_ (mm)	NWT (%)
Amoxicillin	≤0.125	24	≥44	10
Florfenicol	≤2	2	≥46	8
Oxytetracycline	≤0.5	70	≥47	64
Oxolinic acid	≤1	45	≥29	44
Flumequine	≤0.5	39	≥47	41
Enrofloxacin	≤0.125	38	≥49	44

### Correlation of Estimates of the Frequencies of NWT Made from MIC and Disk Data

For the three quinolone agents there was a close agreement between the frequencies of NWT isolates calculated by NRI analysis from the two types (MIC and disk) of test data. For oxolinic acid the frequencies were 45% when MIC data was used and 44% when disk data was used. For flumequine the equivalent frequencies were 39 and 41% for MIC and disk and for enrofloxacin they were 38 and 44%. For amoxicillin, florfenicol, and oxytetracycline there was less agreement. The respective frequencies for amoxicillin being 24 and 10%, for florfenicol they were 2 and 8%, and for oxytetracycline they were 70% when MIC data was used and 64% when disk data was used.

Comparisons of the overall frequencies of NWT phenotypes established by analysis of the MIC and disk diffusion data do not, however, reveal the extent of disagreement on the categorisation of individual isolates as WT or NWT by the two methods. The percentage of isolates where the two methods (MIC and disk) produced different categorisations varied for the six agents. For five, the quinolones, amoxicillin, and oxytetracycline the mean percentage categorical disagreement was 14% (range 18–11%). For florfenicol the categorical disagreement was lower (5%), however, it should be noted that, for this agent, only a small percentage of isolates were categorized as NWT by either method.

The extent of these categorical disagreements suggests that the adoption of both the MIC and disk diffusion methods would result in conflicting results. As the MIC data was the more precise it is strongly suggested that the NWT categorisations obtained using CO_WT_ from the MIC data should be considered as more authoritative.

## Discussion

### Comparison with Other Estimates of Epidemiological Cut-Off Values for *F. psychrophilum* MIC Data

In attempting to compare the CO_WT_ values for MIC data calculated in this work with those that have been suggested by other workers it must be remembered that cut-off values are test-protocol specific. In particular differences in method (agar dilution or broth micro-dilution), and in the media and the incubation conditions used may affect the values of CO_WT_. Differences might also arise when different methods are used to estimate or calculate cut-off values. There are a number of studies (Kum et al., 2008; Del Cerro et al., 2010; Durmaz et al., 2012) where the number of isolates investigated was too small for valid estimates of CO_WT_ to be made. In addition the distribution of MIC values in some studies (Rangdale et al., 1997; Hesami et al., 2010) was too diverse to allow the calculation of a valid CO_WT_. The study carried out under conditions most similar to those used in this work and, therefore, providing the most suitable data for comparison, was that of 387 Danish isolates by Bruun et al. (2000). As in this work, the Danish group employed agar dilution. However, they used 1:7 diluted MH with 5% fetal bovine serum as opposed to the 1.5 diluted MH with no additions that was used in this work. Bruun et al. (2000) did not estimate any cut-off values from their data but it was possible apply NRI retrospectively to the analysis of the observational data they did present. The CO_WT_ calculated from the two studies that used agar dilution data were very similar (**Table 2**). For amoxicillin, florfenicol, and oxytetracycline the CO_WT_ values calculated from the Danish data were identical to those calculated in this work and for oxolinic acid they differed by only one dilution. NRI analysis of the data of Michel et al. (2003) who investigated the florfenicol susceptibility of *F. psychrophilum* using agar dilution calculated a CO_WT_ of ≤2 μg mL^-1^. This value was identical to the values calculated in this work and from the data of Bruun et al. (2000). The similarity of these CO_WT_ values suggests that they may have relevance for the interpretation of MIC values generated using agar dilution protocols in other laboratories.

**Table 2:** Comparison of CO_WT_ (μg mL^-1^) calculated by NRI from the MIC data generated in various studies.

Antibiotic	Method			
	Agar dilution			Broth microdilution
	1:5 diluted MH	1:7 diluted MH + 5% FBS	?	MH 4 g L^-1^
	This work	Bruun et al. (2000)	Michel et al. (2003)	Smith et al. (2016)
	125 isolates	387 isolates	44 isolates	124 isolates
	Chile	Denmark	Europe	Denmark and UK
Amoxicillin	≤0.125	≤0.125	ND	ND
Florfenicol	≤2	≤2	≤2	≤2
Oxytetracycline	≤0.5	≤0.5	ND	≤0.125
Oxolinic acid	≤1	≤0.5	ND	≤0.25

*MH, Mueller-Hinton agar; FBS, fetal bovine serum; ND, not determined.*

Smith et al. (2016) calculated CO_WT_ values by NRI analysis of the MIC values of Danish and UK isolates obtained using a broth micro-dilution method. They employed the CLSI standard protocol for this method (CLSI, 2014a) which recommends the use of diluted MH broth (4 g L^-1^). For florfenicol their CO_WT_ value was identical to that calculated by the agar dilution methods but for oxolinic acid and oxytetracycline their CO_WT_ values were one or two dilutions lower than those calculated in the agar dilution studies (**Table 1**). Henríquez-Núñez et al. (2012) also employed broth micro-dilution to study 40 Chilean isolates. However, their data sets contained too few (<10) WT isolates to allow the calculation of valid CO_WT_ values (Smith and Kronvall, 2015b).

### Comparison of Other Reports of Frequencies of NWT Phenotypes

The Aquatic Animal Health Code of OIE^5^ makes specific recommendations as to the strain collections to be used in investigating the frequencies of those with NWT phenotypes. They recommend that the collections should be sufficiently large and have been obtained from a wide geographic area and that standardized and statistically based methods should be used to generate the cut-off values used to categorize isolates as NWT. In particular, as it is probable that NWT phenotypes would be more common in isolates made from outbreaks that presented therapeutic problems, they suggest that care should be taken to avoid any over representation of isolates from such outbreaks.

Although, probably, none of the studies of *F. psychrophilum* meet all the OIE requirements if they are strictly applied, a comparison of the NWT frequencies reported in those that included at least 40 isolates and for which valid CO_WT_ could be calculated was made (**Table 2**). In making these comparisons it should be noted that the frequencies of NWT phenotypes in different studies should not be influenced by differences in the testing protocols or the CO_WT_ adopted. It would not, however, be surprising for different NWT frequencies to be recorded in studies of isolates recovered from different geographical areas.

In four of the five MIC studies compared (this work, Bruun et al., 2000; Michel et al., 2003; Smith et al., 2016) there was a general agreement in the overall patterns of reduced susceptibility observed (**Table 3**). All reported <2% NWT frequencies for florfenicol. For amoxicillin the frequencies could be characterized as intermediate to low (12–24%) and for oxolinic acid (31–66%) and oxytetracycline (44–70%) they could be characterized as intermediate to high. The frequencies of NWT phenotypes reported for 40 Chilean isolates in a fifth study (Henríquez-Núñez et al., 2012), however, were significantly higher. On the basis of their NRI analysis of their MIC data they reported NWT frequencies of 92.5% for florfenicol, 85% for oxolinic acid, and 90% for oxytetracycline.

**Table 3:** Comparison of the frequencies (%) of NWT phenotypes from the MIC data generated in various studies.

Antibiotic	This work	Henríquez-Núñez et al. (2012)	Smith et al. (2016)	Bruun et al. (2000)	Michel et al. (2003)
	125 isolates	40 isolates	124 isolates	387 isolates	44 isolates
	Chile		Denmark and UK	Denmark	Europe
Amoxicillin	24	ND	14	12	ND
Florfenicol	2	93	0	0	0
Oxytetracycline	70	90	44	69	ND
Oxolinic acid	45	85	31	66	ND

*ND, not determined.*

With respect to studies made on Chilean isolates, both the distribution of MIC values and the NWT frequencies calculated from their data by Henríquez-Núñez et al. (2012) show major differences with those calculated in this work. In the case of florfenicol, these differences are dramatic (**Tables 2** and **3**). We have no data that could provide an explanation of this dramatic difference. When disk data of oxytetracycline and oxolinic acid was analyzed, these differences were also observed, in the distribution of the disk diffusion zone sizes, the cut-off values estimated from them and the NWT frequencies when compared to the reported by Henríquez-Núñez et al. (2012) and only for florfenicol some similarity was detected. The florfenicol zone sizes reported by Henríquez-Núñez et al. (2012) for their WT isolates were all within the range of the WT florfenicol zones obtained in this work. Applying the CO_WT_ they calculated from their florfenicol zone data of ≥55 mm Henríquez-Núñez et al. (2012) reported that the frequency of NWT phenotypes in their isolates was ∼10%. In this work the application of the CO_WT_ ≥ 46 mm to the florfenicol zone sizes obtained calculated an NWT frequency of 8% (**Table 1**). This similarity can be contrasted with the major difference in the frequencies of florfenicol NWT phenotypes established from the analysis of the respective MIC data in these two studies. From an analysis of their MIC data Henríquez-Núñez et al. (2012) reported an NWT frequency of 92.5% whereas the frequency established in this work was 2% (**Table 1**).

### Clinical Significance of NWT Phenotypes

Epidemiological cut-off values are set from a consideration of *in vitro* susceptibility data and as a result they can have no inherent clinical significance. However, Smith (2012) has argued that these cut-off values do provide guidance to susceptibility testing laboratories as to how to report the meaning of the data they record. If a clinical isolate is classified as WT, they can state that susceptibility testing has revealed no reason why therapy should not be initiated. If a clinical isolate is classified as NWT they can state that the isolate manifests a reduced susceptibility and that this reduction may be sufficient to compromise therapy and that susceptibility testing suggests that the initiation of therapy with this agent may be imprudent (Smith, 2008).

There are two approaches toward producing interpretive criteria of more direct clinical relevance. The first is to collect a large database of the clinical outcomes of on-farm therapies and to attempt to correlate these outcomes with the MIC values of the isolates obtained from them. The second is to perform experiments similar to those reported by Bruun et al. (2003). They studied the success of a standard oxytetracycline in treating laboratory-based challenges with *F. psychrophilum* isolates of various susceptibilities. They demonstrated that their therapies successfully controlled infections with a strain with a MIC of 0.25 μg mL^-1^ but had greatly reduced success with a strain that had a MIC of 4 μg mL^-1^. These experimental observations suggest that it would be reasonable and prudent for a laboratory to report isolates with a MIC of ≥0.5 μg mL^-1^ for oxytetracycline as probably clinically resistant to this agent.

## Conclusion

The data obtained in this work demonstrated that the agar dilution susceptibility test protocol employing dilute (1:5) MH media was a suitable method for the analysis of the susceptibility of *F. psychrophilum* isolates. Under the conditions specified by the protocol all 125 isolates of *F. psychrophilum* investigated were capable of sufficient growth to facilitate the unambiguous determination of their MIC values. Application of NRI analysis demonstrated that MIC data sets generated by this protocol for six agents were sufficiently precise that valid laboratory-specific CO_WT_ could be calculated from them. These CO_WT_ values were either identical or within one dilution of those calculated for other data sets generated by agar dilution protocols (Bruun et al., 2000; Michel et al., 2003). It is, therefore, possible that these cut-off values might serve as general laboratory-independent interpretive criteria.

The data generated by the disk diffusion protocol used in this work were shown to have low precision. On this basis, we agree with the suggestion of Henríquez-Núñez et al. (2012) that this method is not suitable for investigating the susceptibility of *F. psychrophilum* isolates.

## Author Contributions

CM designed the study, cultured all bacterial isolates, performed all antimicrobial susceptibility assays, drafted the manuscript, and is the corresponding author and primary contact during the manuscript submission, review, and publication process. PS performed the NRI analyses of inhibition zone and MIC data as well as contributed significantly to the drafting, revisions, and interpretation of data. RR contributed making Tables and Figures and draft editing. SC-L helped with drafting and revisions of manuscript. JV helped to discuss the results of NRI analysis. All authors have made intellectual contribution to the work, and approved it for publication.

## Conflict of Interest Statement

The authors declare that the research was conducted in the absence of any commercial or financial relationships that could be construed as a potential conflict of interest.

## Footnotes

1 http://www.oie.int/international-standard-setting/aquatic-code/access-online

2 http://rdp.cme.msu.edu/

3 http://www.bioscand.se/nri/

4 http://www.eucast.org/mic_distributions_and_ecoffs/

5 http://www.oie.int/international-standard-setting/aquatic-code/access-online
